# Insufficient radiofrequency ablation promotes epithelial-mesenchymal transition of hepatocellular carcinoma cells through Akt and ERK signaling pathways

**DOI:** 10.1186/1479-5876-11-273

**Published:** 2013-10-29

**Authors:** Shuying Dong, Jian Kong, Fandong Kong, Jinge Kong, Jun Gao, Shan Ke, Shaohong Wang, Xuemei Ding, Wenbing Sun, Lemin Zheng

**Affiliations:** 1 Department of Hepatobiliary Surgery, Beijing Chaoyang Hospital, Capital Medical University, Beijing, 100043, China; 2 The Institute of Cardiovascular Sciences and Institute of Systems Biomedicine, School of Basic Medical Sciences, Peking University Health Science Center, Key Laboratory of Molecular Cardiovascular Sciences of Education Ministry, and Key Laboratory of Cardiovascular Molecular Biology and Regulatory Peptides of Health Ministry, Beijing 100191, China; 3 The Neuroscience Research Institute & Department of Neurobiology, School of Basic Medical Sciences, Key Laboratory for Neuroscience, Ministry of Education/National Health and Family Planning Commission, Peking University, Beijing 100191, China

**Keywords:** Insufficient radiofrequency ablation, Epithelial-mesenchymal transition, Hepatocellular carcinoma, Metastasis

## Abstract

**Background:**

Residual tumor progression after insufficient radiofrequency ablation (RFA) has been recently reported. However, whether epithelial-mesenchymal transition (EMT), which is a key process that drives cancer metastasis, is involved in the tumor progression after insufficient RFA is not well understood.

**Methods:**

Human hepatocellular carcinoma (HCC) cell lines SMMC7721 and Huh7 were used. Insufficient RFA was simulated using a water bath (47°C 5 min, 10 min, 15 min, 20 min and 25 min gradually). MTT assay was used to evaluate the proliferation of HCC cells *in vitro*. Migration and invasion of HCC cells were determined by transwell assay. The molecular changes in HCC cells after insufficient RFA were evaluated by western blot. LY294002 and PD98059 were used to treat HCC cells. An ectopic nude mice model and a tail vein metastatic assay were used to evaluate the growth and metastatic potential of SMMC7721 cells *in vivo* after insufficient RFA.

**Results:**

SMMC7721 and Huh7 cells after insufficient RFA (named as SMMC7721-H and Huh7-H respectively) exhibited enhanced proliferation, migration and invasion (6.4% and 23.6%, 33.2% and 66.1%, and 44.1% and 57.4% increase respectively) *in vitro*. Molecular changes of EMT were observed in SMMC7721-H and Huh7-H cells. LY294002 and PD98059 inhibited the EMT of SMMC7721-H and Huh7-H cells. SMMC7721-H cells also exhibited larger tumor size (1440.8 ± 250.3 mm^3^ versus 1048.56 ± 227.6 mm^3^) and more lung metastasis (97.4% increase) than SMMC7721 cells *in vivo*. Higher expression of PCNA, N-cadherin and MMP-2 and MMP-9, was also observed in SMMC7721-H tumors.

**Conclusions:**

Insufficient RFA could directly promote the invasiveness and metastasis of HCC cells. Insufficient RFA may promote the EMT of HCC cells through Akt and ERK signaling pathways.

## Background

Hepatocellular carcinoma (HCC) is the fifth most common cancer in men and the seventh in women worldwide [1]. Radiofrequency ablation (RFA) is one of the treatments for HCC and is now widely used for curative strategies [2]. However, for the RFA procedure to be considered technically successful, the tumor and a safety margin of at least 5 mm of normal hepatic tissue must be completely included in the ablation zone [3], therefore the major problem with RFA is its difficulty in achieving complete tumor destruction [4].

Residual tumor progression after insufficient RFA has been recently reported and two possible mechanisms also have been proposed. RFA may alter tumor microenvironment to enhance the outgrowth of residual tumor cells. RFA could accelerate perinecrotic outgrowth of colorectal liver metastases in a hypoxia-dependent manner [4,5]. Another study showed that thermal ablation promoted the progression of micrometastases to form macroscopically detectable neoplasms in treated regenerating liver through an increased expression of vascular endothelial growth factor (VEGF) and fibroblast growth factor-2 (FGF-2) adjacent to the treatment site [6]. Our previous study also showed that tumor-associated endothelial cells after insufficient RFA exhibited enhanced angiogenesis and promoted invasiveness of residual HCC [7]. Alternatively, RFA could directly influence tumor cells to promote progression of residual tumor. Our previous studies demonstrated that HCC cells after insufficient RFA induced angiogenesis via hypoxia inducer factor (HIF-1) α/ VEGFA *in vitro*[8], and insufficient RFA could facilitate the growth and metastasis of residual hepatic VX2 carcinoma owing to the induction of over-expression of PCNA, VEGF and MMP-9 [9]. Another study also indicated that insufficient RFA may induce further malignant transformation of HCC [10]. However, rapid progression of residual tumor after insufficient RFA is a complex process and further mechanisms need to be elucidated. Metastases, termed the invasion-metastasis cascade, involve dissemination of cancer cells to anatomically distant organ sites and their subsequent adaptation to foreign tissue microenvironments [11], which 90% of mortality from cancer is attributable to [12]. Whether insufficient RFA could directly promote invasion-metastasis of residual HCC cells and the mechanisms involved in the process have not been clearly determined.

Epithelial-mesenchymal transition (EMT) is a key process that drives cancer metastasis, and it is characterized by loss of the epithelial marker, increased expression of the mesenchymal marker, and enhanced migratory and invasive behaviors [13,14]. Characteristic down-regulation of E-cadherin is regarded as the key step to EMT. HCCs with EMT features consistently exhibit more venous invasion, metastases, and a poorer prognosis than those without EMT characteristics [15]. Whether insufficient RFA directly induces the EMT of residual HCC cells and further promotes the metastasis remains unclear.

In the present study, we investigated the morphological changes, cell growth, migration and invasion of HCC cell lines (SMMC7721 and Huh7) after insufficient RFA *in vitro*. Furthermore, we analyzed the changes of epithelial and mesenchymal markers, and Akt and ERK1/2 signaling pathways involved in the process in HCC cells after insufficient RFA. We also performed *in vivo* experiments to study the growth and metastasis of HCC cells after insufficient RFA in a BALB/c nu/nu mice model.

## Methods

### Cell culture

Established human HCC cell lines, SMMC7721 and Huh7 were from the American Type Culture Collection (ATCC; Manassas, VA, USA). All cells were maintained in high-glucose Dulbecco’s modified Eagle medium (DMEM) supplement with 10% fetal bovine serum (FBS), 100 U/ml penicillin and 100 μg/ml streptomycin (Life Technologies, Cergy Pontoise, France) in a humidified atmosphere of 5% CO_2_ at 37°C.

### Chemicals and antibodies

LY294002 and PD98059 were purchased from Beyotime (Jiangsu, China). Antibodies with specificity for the phosphorylated forms of Akt and ERK1/2 were purchased from Cell signaling (Beverly, CA, USA). Antibodies recognizing E-cadherin, N-cadherin, vimentin, snail and α-SMA were bought from Abcam (Cambridge, TX, USA). Antibodies recognizing β-actin, MMP-2 and MMP-9 antibodies were obtained from Santa Cruz (Dallas, TX, USA).

### Heat treatment

Insufficient RFA was simulated *in vitro* as described before [8]. Briefly, SMMC7721 or Huh7 cells were seeded into the 6-well plates (5 × 10^4^ cells/well). After 24 h, the plates were sealed and submerged in a water bath set to 47°C for 5 min. Thereafter, cells were allowed to recover, and when the surviving populations reached 80% confluence, cells were propagated into the 6-well plates and exposed to above heat treatment for 10 min. Then the process was repeated and cells were sequentially exposed to above heat treatment for 15 min, 20 min and 25 min. Cells survived from the treatment were designated as SMMC7721-H and Huh7-H respectively. The morphological characteristics of HCC cells were observed by microscopy (Olympus, Tokyo, Japan).

### Proliferation assay

Cell proliferation was analyzed using the 3-(4, 5-dimethylthiazol-2-yl)-2, 5-diphenyltetrazolium bromide (MTT) assay. Briefly, HCC cells were cultured in 96-well plates at a concentration of 3 × 10^3^ cells/well, and incubated for 24 h, 48 h, or 72 h. MTT solution was added to each well at a final concentration of 0.5 mg/ml and incubated for 4 h. At the end of incubation, formazan crystals resulting from MTT reduction were dissolved by addition of 150 μl dimethyl sulfoxide per well. The absorbance was measured at 570 nm using an automated ELISA plate reader.

### Colony formation assay

HCC cells were seeded into 6-well dishes at a concentration of 1 × 10^3^ cells/well and allowed to grow in complete medium for 2 weeks. The colonies obtained were washed with PBS and fixed in 4% paraformaldehyde for 20 min at room temperature and then washed with PBS followed by staining with crystal violet. The colonies were counted and compared with untreated cells.

### Migration and invasion assay

Quantitative cell migration assays were performed using a modified Boyden chamber (Costar-Corning, New York, USA) with 8.0-μm pore polycarbonate filter inserts in 24-well plates as described previously. Briefly, the lower chamber was filled with DMEM with 10% FBS, and HCC cells (5 × 10^4^ cells/well) in serum-free medium were added into the upper chamber. The cells were allowed to migrate for 24 h at 37°C. The non-migrated cells were removed from the upper surface of the membrane by scraping with a cotton swab, and the migrating cells were fixed with methanol, stained with crystal violet (Beyotime, Nantong, China) and photographed under an inverted fluorescence microscope (Olympus IX51) equipped with an Olympus Qcolor 3 digital camera (Olympus). Migration was assessed by counting the number of stained cells from 10 random fields at × 200 magnification. Cell invasion assay was performed similarly, except that transwell inserts were matrigel-coated.

### Western blot

HCC cells were lysed with lysis buffer [150 mM NaCl, 50 mM Tris–HCl (pH 8.0), 0.1% SDS, 1% Triton X-100] containing protease and phosphatase inhibitor (1 mM Phenylmethanesulfonyl fluoride and 1 mM Sodium orthovanadate). Cell lysate protein content was determined using a Bicinchoninic acid (BCA) protein assay kit. Equivalent amounts of whole cell extracts were subjected to SDS-PAGE and transferred to nitrocellulose membranes. The membranes were blocked with 5% non-fat milk for 2 h and then incubated with respective primary antibody overnight at 4°C followed by the incubation with the appropriate HRP-conjugated secondary antibody for 1.5 h at room temperature. Blots were visualized with an ECL detection kit (Pierce, USA) and analyzed using Quantity One 1-D Analysis Software (Bio-Rad, Hercules, USA).

### Inhibitors

LY294002 or PD98059 was used to inhibit the expression of p-Akt or p-ERK1/2 in HCC cells. Briefly, LY294002 or PD98059 was added to the culture media of HCC cells at a final concentration of 25 μM or 50 μM, after 24 h, cell lysate protein was collected, and western blot was conducted. In the migration and invasion assays, LY294002 (25 μM) or PD98059 (25 μM) was added to the upper chamber, and after 24 h the chambers were collected.

### Animals

Male BALB/c nu/nu mice (4–6 weeks old) were obtained from Vital River Laboratories (Beijing, China) and maintained under standard pathogen-free conditions. The animal welfare guidelines for the care and use of laboratory animals were approved by the Animal Care Committee of Capital Medical University (Beijing, China).

### Xenograft assays

SMMC7721 cells (5 × 10^6^ cells) were suspended in 200 μl serum-free DMEM and matrigel (1:1) and then injected subcutaneously into the upper right flank region of 12 nude mice. Tumor size was measured with a caliper rule every 3 days. The tumor volume was estimated with the formula “a × b^2^ × 0.5”, in which a represented the longest and b the shortest radius of the tumor in millimeters. At the end of the experiments, mice were euthanized, blood samples were collected via cardiac puncture, and tumor tissues were removed for fixation in the 4% paraformaldehyde for histologic examination and immunohistochemical staining.

### Tail vein metastatic assays

HCC cells (1 × 10^6^ cells) were suspended in 100 μl PBS and injected through tail vein. Four weeks after the injection, the mice were sacrificed and the lung tissues were isolated. After counting the number of visible tumors on lung surface, the lung tissues were made into serial sections before HE staining and observed under a light microscope.

### Immunocytochemistry

Tissues were fixed in 4% paraformaldehyde and subsequently embedded in paraffin. Paraffin-embedded tissue sections were cut into standard 6 μm sections, deparaffinaged in xylene and rehydrated through graded alcohol solutions. Antigen retrieval was performed 10 min at 92°C in EDTA (10 mM, pH 8.0) in a water bath. Endogenous peroxidases were inactivated by immersing the sections in 0.3% hydrogen peroxide for 12 min. The sections were blocked with 5% goat serum for 60 min at 37°C. The slides were incubated with primary antibodies for overnight at 4°C. Next, the slides were treated with appropriate HRP-conjugated secondary antibodies for 40 min at 37°C and then developed with 3,3’-diaminobenzidine. Finally, the slides were counterstained with hematoxylin and mounted. The slides were examined with Nikon Eclipse Ti microscope under a × 200 objective.

### Statistical analysis

All values are expressed as the mean ± SEM. The data were analyzed using Student’s t test or the ANOVA test. A P value of <0.05 was considered statistically significant. GraphPad Prism (GraphPad Software Inc., San Diego, California, USA) was used for these analyses.

## Results

### Insufficient RFA promoted HCC cells proliferation, migration and invasion

**Figure 1:**
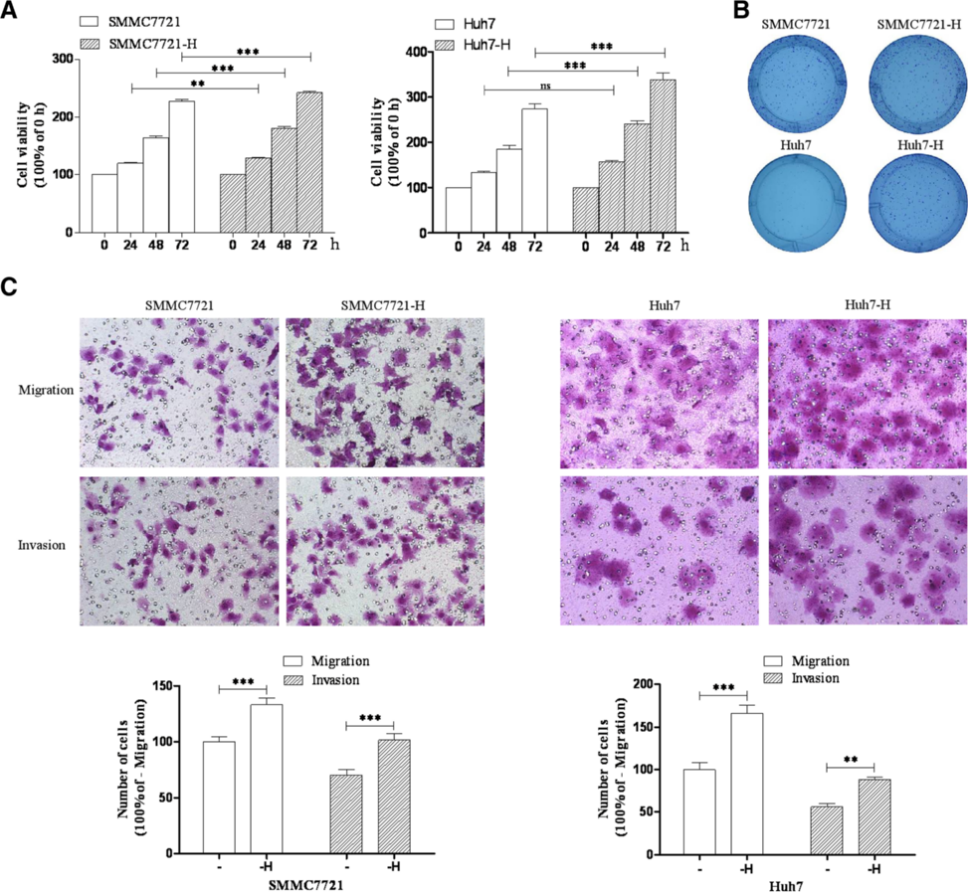
**Insufficient RFA promoted the proliferation, colony formation, migration and invasion of HCC cells.** SMMC7721 and Huh7 cells were treated with insufficient RFA (47°C 5 min, 10 min, 15 min, 20 min and 25 min) gradually. Residual SMMC7721 and Huh7 (named as SMMC7721-H and Huh7-H respectively) cells were collected and used for the next experiments. **(A)** Proliferation rate of SMMC7721, SMMC7721-H, Huh7 and Huh7-H was evaluated by MTT assay. Error bars represent the SEM of data obtained in five independent experiments. **(B)** Colony formation abilities of SMMC7721, SMMC7721-H, Huh7 and Huh7-H cells were assessed. Error bars represent the SEM of data obtained in three independent experiments. **(C)** Migration and invasion of SMMC7721, SMMC7721-H, Huh7 and Huh7-H cells were shown. Error bars represent the SEM of data obtained in three independent experiments. P value <0.05 was considered statistically significant; ***p < 0.001, **p < 0.01.

To evaluate the effect of insufficient RFA on HCC cells, SMMC7721 and Huh7 cells were treated with heat treatment (47°C in a water bath) for 5 min, 10 min, 15 min, 20 min and 25 min gradually as described previously [8]. Three independent SMMC7721-H or Huh7-H cell lines have ever been developed, and biological behavior of each SMMC7721-H or Huh7-H cell line was similar. The results of one of each were shown. SMMC7721-H exhibited higher proliferation rate compared with SMMC-7721 at 24 h, 48 h, and 72 h (7.6%, 9.9% and 6.4% increase respectively) (Figure 1A). To determine the long term growth ability, HCC cells were allowed to grow for 2 weeks. SMMC7721-H cells had a higher number of colonies in comparing with SMMC7721 cells (Figure 1B). SMMC7721-H cells also displayed enhanced migration and invasion abilities (33.2% and 44.1% increase respectively) compared with SMMC7721 cells (Figure 1C). Similar patterns of cell proliferation, migration and invasion were also found in Huh7-H and Huh7 cells (Figure 1).

### Insufficient RFA promoted EMT of HCC cells

**Figure 2:**
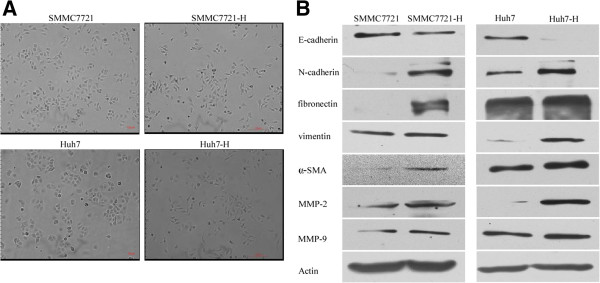
**Insufficient RFA induced EMT in HCC cells. (A)** The morphological changes of SMMC7721 and Huh7 cells after insufficient RFA were displayed. **(B)** Cell extracts were subjected to western blot analysis and expression of E-cadherin, N-cadherin, fibronectin, vimentin, α-SMA, MMP-2 and MMP-9 were detected. Actin served as loading control.

Interestingly, we found that SMMC7721-H and Huh7-H displayed a spindle shape with less cell-cell adhesion and increased formation of pseudopodia (Figure 2A). To evaluate whether EMT had occurred in SMMC7721-H and Huh7-H cells, EMT markers were examined. Western blot showed significant reduction in E-cadherin expression and up-regulation of N-cadherin, vimentin, α-SMA, fibronectin, MMP-2 and MMP-9 (Figure 2B).

### Insufficient RFA promoted EMT of HCC cells through Akt and ERK1/2 signaling pathways

**Figure 3:**
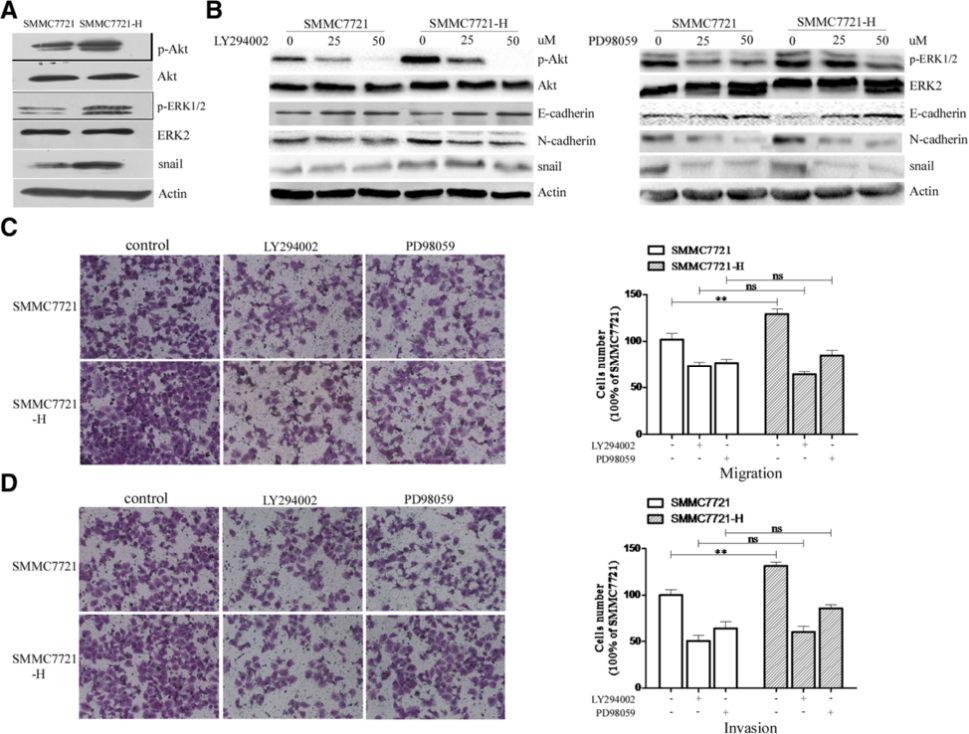
**Insufficient RFA promoted EMT of SMMC7721 cells through Akt and ERK1/2 signaling pathways. (A)** The expression of p-Akt, Akt, p-ERK1/2, ERK2 and snail in SMMC7721 and SMMC7721-H cells were assessed by western blot. **(B)** LY294002 or PD98059 was used to treat HCC cells, and western blot was used to determined the expression of p-Akt, Akt, p-ERK1/2, ERK2, E-cadherin, N-cadherin, and snail. **(C-D)** LY294002 or PD98059 was used to treat SMMC7721 and SMMC7721-H cells, and migration **(C)** and invasion **(D)** of SMMC7721 and SMMC7721-H were evaluated. Error bars represent the SEM of data obtained in three independent experiments. P value <0.05 was considered statistically significant; ***p < 0.001, **p < 0.01, ns, no significance.

To explore the signaling mechanisms involved in the EMT of HCC cells after insufficient RFA, we tested Akt and ERK1/2 signaling pathways. SMMC7721-H showed significantly increased expression of p-Akt and p-ERK1/2 compared with SMMC7721 (Figure 3A). Furthermore, an up-regulation of the transcription factor snail was also detected in SMMC7721-H (Figure 3A). PI3K/Akt inhibitor LY294002, or ERK1/2 inhibitor PD98059 significantly suppressed the expression of p-Akt or p-ERK1/2 in SMMC7721 and SMMC7721-H cells respectively, also inhibited the expression of N-cadherin and snail, and increased the expression of E-cadherin (Figure 3B). LY294002 or PD98059 also suppressed the migratory and invasive ability of SMMC7721 and SMMC7721-H (Figure 3C and D). The significant difference of migratory and invasive ability of SMMC7721 and SMMC7721-H cells was also eliminated after LY294002 or PD98059 was used (Figure 3C and D). Similar results were also found in Huh7 and Huh7-H cells (Additional file 1).

### Insufficient RFA enhanced the growth of HCC cells *in vivo*

**Figure 4:**
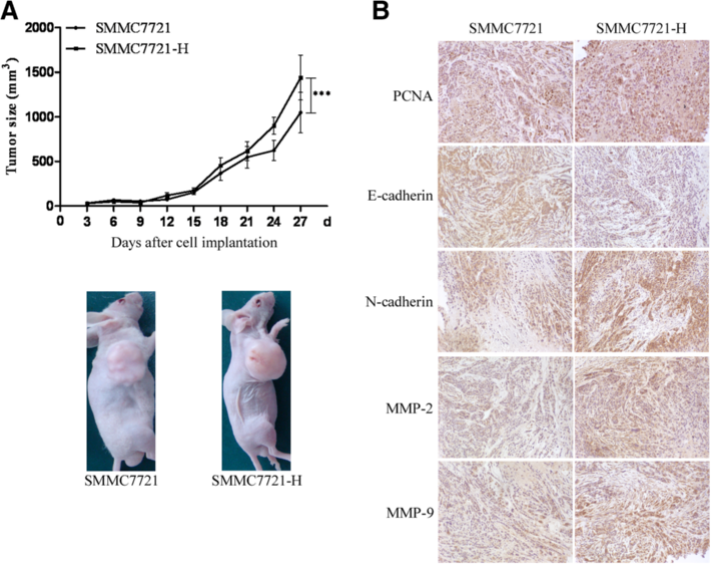
**Insufficient RFA promoted the growth of HCC cells *in vivo*.** SMMC7721 and SMMC7721-H cells were injected subcutaneously into the upper right flank region of nude mice and tumor volume was measured. **(A)** Tumor volume was measured with a caliper rule every 3 days. Data were presented as the mean tumor volumes of mice. Representative images of tumor volume were also exhibited. **(B)** Tumor sections were stained with PCNA, MMP-2, MMP-9, E-cadherin and N-cadherin. Representive images of the immunohistochemistry assay were shown (× 200).

To examine the effects of insufficient RFA on tumor growth *in vivo*, we evaluated the effect in a SMMC7721 ectopic HCC model. SMMC7721-H cells showed increased tumor volume compared with SMMC7721 cells (1440.8 ± 250.3 mm^3^ versus 1048.56 ± 227.6 mm^3^) (Figure 4A). Significant increases of cell proliferation were observed by PCNA in SMMC7721-H tumors. In addition, SMMC7721-H tumors showed decreased expression of E-cadherin and increased expression of N-cadherin, MMP-2 and MMP-9 compared with SMMC7721 tumors (Figure 4B). However, there were no apparent changes in body weight in the mice (Additional file 2A).

### HCC cells exhibited enhanced metastatic ability *in vivo* after insufficient RFA

**Figure 5:**
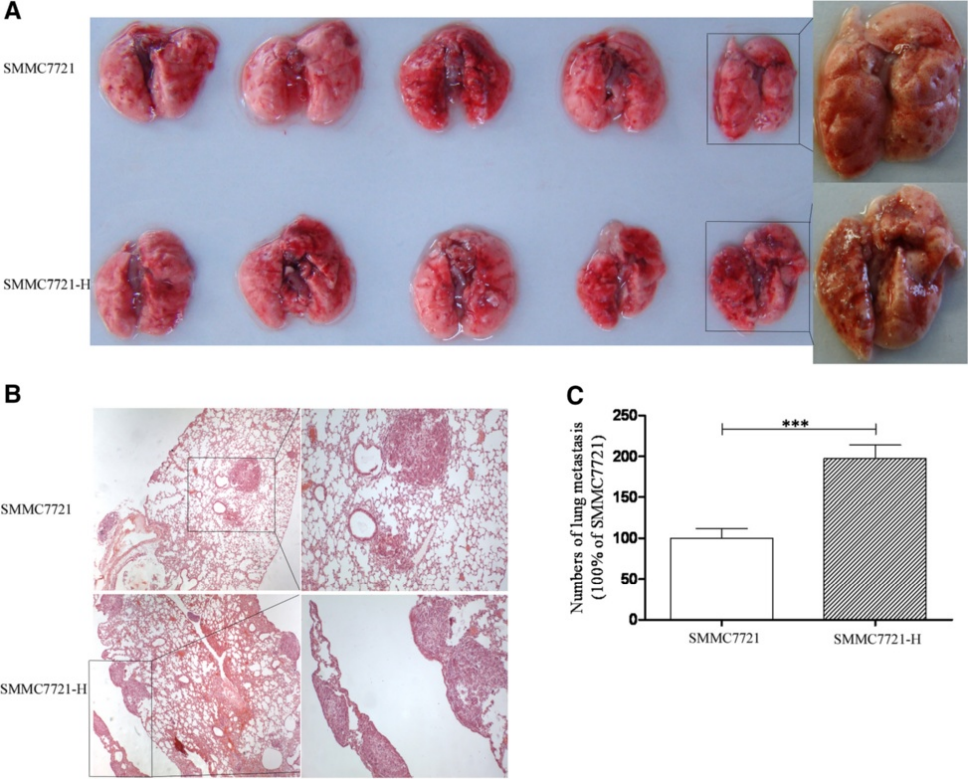
**HCC cells after insufficient RFA exhibited enhanced metastatic ability *in vivo*.** SMMC7721 and SMMC7721-H cells were injected through tail vein of mice. **(A)** Lung metastasis was assessed. **(B)** The lung tissues were sectioned serially and stained with HE. Representative images of HE were shown (× 200). **(C)** The numbers of metastatic tumors were counted. Error bars represent the SEM of data obtained in three independent experiments. P value <0.05 was considered statistically significant; ***p < 0.001.

To determine the effects of insufficient RFA on the *in vivo* metastasis of SMMC7721 cells, a tail vein metastasis assay was used. The extent of the metastatic tumors on the surface of the lung was significantly increased (97.4% increase) in mice receiving SMMC7721-H cells compared with SMMC7721 cells (Figure 5A). The lung tissues were sectioned serially and HE staining also confirmed the results above (Figure 5B and C). However, there were no apparent changes in body weight in the mice (Additional file 2B).

## Discussion

RFA is safe and more effective than resection for very early HCC and in the presence of two or three nodules <3 cm, however, its ability to obtain complete and sustained tumor necrosis is less predictable [16]. So to further elucidate the biological behavior of residual HCC, involved mechanisms after insufficient RFA is important to improve prognosis of HCC patients. In the present study, we demonstrated that insufficient RFA promoted the growth, migration and invasive potential of HCC cells. Furthermore, enhanced migration and invasion of HCC cells after insufficient RFA were associated with EMT. In addition, rapid growth and enhanced metastasis of HCC cells after insufficient RFA *in vivo* further confirmed the results *in vitro*. Our results have demonstrated that EMT plays an important role in enhancing invasiveness and metastasis of HCC cells after insufficient RFA.

Our previous study elucidated that one sub-line selected from HepG2 cells after insufficient RFA (47°C 10 min) exhibited more rapid proliferation rate [8]. Although in the present study SMMC7721 and Huh7 cells were treated with insufficient RFA gradually (47°C 5 min, 10 min, 15 min, 20 min, and 25 min), the surviving SMMC7721-H and Huh7-H cells also showed higher proliferation rate compared with SMMC7721 and Huh7 cells respectively. Interestingly, in the present study, SMMC7721 and Huh7 cells after insufficient RFA displayed a spindle shape with less cell-cell adhesion and increased formation of pseudopodia. So we inferred that insufficient RFA may also induce the genomic instability of HCC cells. However, the mechanisms involved in the process have not been elucidated and need to be studied in the further.

Metastasis is a multistage process that requires cancer cells to escape from the primary tumor, survive in the circulation, seed at distant sites and grow [17]. Metastasis has also always been a bottleneck in tumor prognosis and therapy [18]. Metastasis, both intrahepatic and extrahepatic, is of particular concern and occurs in more than half of HCC cases [19]. Our previous study suggested that tumor-associated endothelial cells after insufficient RFA could promote invasiveness of residual HCC cells *in vitro*[7]. Whether insufficient RFA could enhance invasive potential of HCC cells has not been determined. In this study, we found that SMMC7721 and Huh7 cells after insufficient RFA also exhibited enhanced migration and invasive potential. The EMT appears to be essential for cancerous cells to acquire the capability of migration and invasion and is a key driver to tumor cell translocation [20]. EMT is also a process whereby cells change from cobble-stone shapes that exhibit tight cell-cell contact into spindle-shape fibroblast-like shapes that lose cell-cell contact and cell polarity [21-23]. The morphological changes of SMMC7721-H and Huh7-H cells were consistent with the characteristics of EMT. Down-regulation of E-cadherin and up-regulation of N-cadherin, vimentin, α-SMA, and fibronectin further confirmed that EMT occurred in HCC cells after insufficient RFA. Recently, Yoshida S et al. also demonstrated that sub-lethal heat treatment promoted EMT and enhanced the malignant potential of HCC, which was partly consistent with our results [24]. The tail vein metastasis assay also showed that HCC cells after insufficient RFA exhibited enhanced pulmonary metastasis ability, which may further support our results *in vivo*. The results also showed that HCC cells after insufficient RFA had enhanced abilities of surviving in the circulation, colonization and outgrowth within a secondary organ, in which mesenchymal to epithelial transition (MET) plays a key role [25]. The complex mechanisms involved in the metastasis of HCC cells after insufficient RFA still need to be determined. Furthermore, we examined the growth of HCC cells after insufficient RFA *in vivo*. The expression of PCNA and N-cadherin was higher and the expression of E-cadherin was lower in SMMC7721-H cells than SMMC7721 cells, which was consistent with the results *in vitro*.

Lang BJ et al. reported that heat stress enhanced cell migration in both the lung A549, and breast MDA-MB-468 human adenocarcinoma cell lines, with A549 cells also undergoing a partial EMT [26]. The heat stress used in their study was 42°C 30 min, and the temperature was 47°C 5 min, 10 min, 15 min, 20 min and 25 min in our study, however, the results was partly consistent. Although Lang BJ et al. demonstrated that heat stress promoted cell migration independent of heat shock factor 1, the mechanisms involved in the process had not been further determined. Recently, Akt and ERK signaling pathways have been reported to play a key role in the EMT of cancers. Hepatitis B virus X protein repressed miRNA-148a to enhance tumorigenesis through Akt and ERK mediating EMT of HCC [27]. ERK/Akt also regulated EZH2 and E-cadherin to influence the EMT of cancer [28]. TMPRSS4 and TAAC3 promoted EMT through the activation of PI3K/Akt and ERK signaling pathways [29,30]. Akt and ERK signaling pathways also mediated HGF [31,32], TGF-β [33,34], and EGFR [35,36] inducing EMT. In our study, HCC cells after insufficient RFA exhibited higher expression of p-Akt and p-ERK1/2, and PI3K inhibitor, LY294002, and ERK inhibitor, PD98059, significantly inhibited the expression of p-Akt and p-ERK1/2 respectively. LY294002 and PD98059 suppressed the migratory and invasive abilities of SMMC7721-H and Huh7-H cells, and also inhibited the higher expression of N-cadherin, fibronectin, vimentin, α-SMA and snail in SMMC7721-H and Huh7-H cells. Our results suggested that insufficient RFA may induce the EMT of HCC cells through Akt and ERK signaling pathways.

## Conclusions

Our results suggest that insufficient RFA could directly promote the invasiveness and metastasis of HCC cells. Insufficient RFA may promote the EMT of HCC cells through Akt and ERK signaling pathways.

## Competing interests

The authors declare that they have no competing interests.

## Authors’ contributions

SYD, JK and FDK carried out the experiments and drafted the manuscript. JGK and SHW participated in the sequence alignment. LMZ and WBS conceived the study and coordination and helped to draft the manuscript. JG, SK, and XMD participated in the design of the study. All authors read and approved the final manuscript.

## Supplementary Material

Additional file 1 **Insufficient RFA promoted EMT of Huh7 cells through Akt and ERK1/2 signaling pathways.** (A) The expression of p-Akt, Akt, p-ERK1/2, ERK2 and snail in Huh7 and Huh7-H cells were assessed by western blot. (B) LY294002 or PD98059 was used to treat HCC cells, and western blot was used to determine the expression of p-Akt, Akt, p-ERK1/2, ERK2, E-cadherin, N-cadherin, and snail. (C-D) LY294002 or PD98059 was used to treat Huh7 and Huh7-H cells, and migration (C) and invasion (D) of Huh7 and Huh7-H were evaluated. Error bars represent the SEM of data obtained in three independent experiments. P value <0.05 was considered statistically significant; ***p < 0.001, **p < 0.01, ns, no significance. Click here for file

Additional file 2 **Curves of mice body weight in the xenograft assays and tail vein metastatic assays.** Mice body weight in the xenograft assays (A) and tain vein metastatic assays (B) were shown. Click here for file
