# Supplementary material for: Insufficient radiofrequency ablation promotes epithelial-mesenchymal transition of hepatocellular carcinoma cells through Akt and ERK signaling pathways
Source: J Transl Med. 2013 Oct 29;11:273. doi: 10.1186/1479-5876-11-273 (PMC3842745; doi:10.1186/1479-5876-11-273)

**Additional file 1: Insufficient RFA promoted EMT of Huh7 cells through Akt and ERK1/2 signaling pathways.** (A) The expression of p-Akt, Akt, p-ERK1/2, ERK2 and snail in Huh7 and Huh7-H cells were assessed by western blot. (B) LY294002 or PD98059 was used to treat HCC cells, and western blot was used to determine the expression of p-Akt, Akt, p-ERK1/2, ERK2, E-cadherin, N-cadherin, and snail. (C-D) LY294002 or PD98059 was used to treat Huh7 and Huh7-H cells, and migration (C) and invasion (D) of Huh7 and Huh7-H were evaluated. Error bars represent the SEM of data obtained in three independent experiments. P value <0.05 was considered statistically significant; ***p<0.001, **p<0.01, ns, no significance.


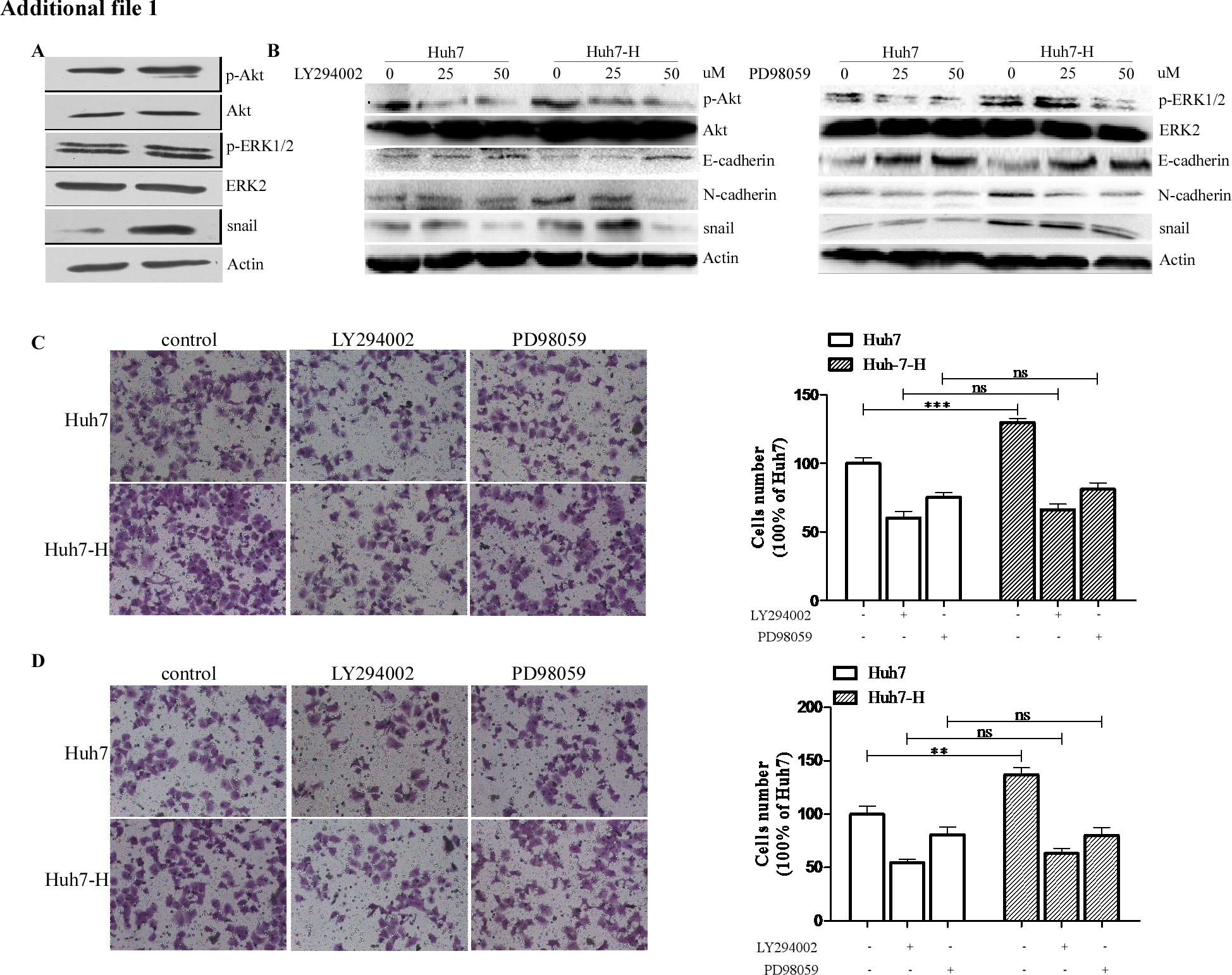

Supplement: Additional file 1 — Insufficient RFA promoted EMT of Huh7 cells through Akt and ERK1/2 signaling pathways. (A) The expression of p-Akt, Akt, p-ERK1/2, ERK2 and snail in Huh7 and Huh7-H cells were assessed by western blot. (B) LY294002 or PD98059 was used to treat HCC cells, and western blot was used to determine the expression of p-Akt, Akt, p-ERK1/2, ERK2, E-cadherin, N-cadherin, and snail. (C-D) LY294002 or PD98059 was used to treat Huh7 and Huh7-H cells, and migration (C) and invasion (D) of Huh7 and Huh7-H were evaluated. Error bars represent the SEM of data obtained in three independent experiments. P value <0.05 was considered statistically significant; ***p < 0.001, **p < 0.01, ns, no significance. [file 1479-5876-11-273-S1.doc]
