# Supplementary material for: Insufficient radiofrequency ablation promotes epithelial-mesenchymal transition of hepatocellular carcinoma cells through Akt and ERK signaling pathways
Source: J Transl Med. 2013 Oct 29;11:273. doi: 10.1186/1479-5876-11-273 (PMC3842745; doi:10.1186/1479-5876-11-273)

**Additional file 2: Curves of mice body weight in the xenograft assays and tail vein metastatic assays.** Mice body weight in the xenograft assays (A) and tain vein metastatic assays (B) were shown.


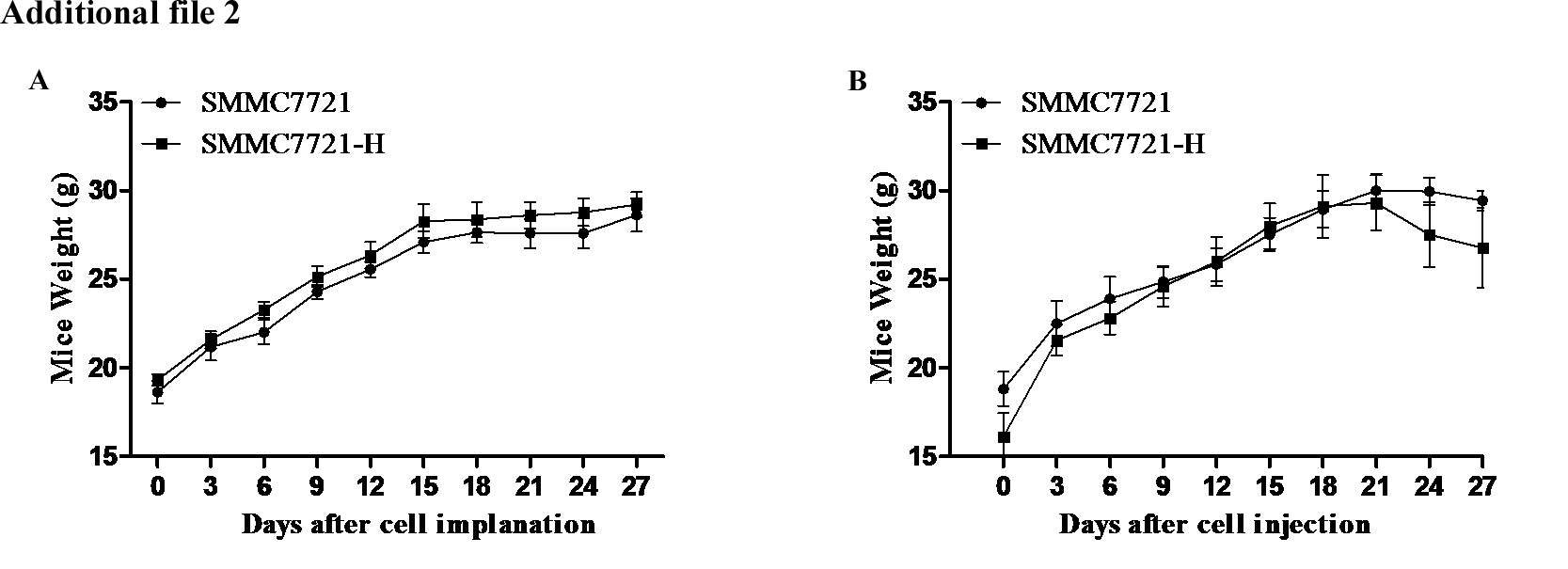

Supplement: Additional file 2 — Curves of mice body weight in the xenograft assays and tail vein metastatic assays. Mice body weight in the xenograft assays (A) and tain vein metastatic assays (B) were shown. [file 1479-5876-11-273-S2.doc]
